# A Dual-Pump Switchable Irrigation System for Hemostasis under Gel Immersion during Water Pressure Duodenal Endoscopic Submucosal Dissection

**DOI:** 10.1055/a-2976-5638

**Published:** 2026-10-15

**Authors:** Gen Kitahara, Takuya Wada, Hisatomo Ikehara, Chika Kusano

**Affiliations:** 1 Department of Gastroenterology 38088 Kitasato University School of MedicineSagamiharaJapan

**Keywords:** endoscopy upper GI tract, endoscopic resection (ESD, EMRc, ...), non-variceal bleeding, diagnosis and imaging (inc chromoendoscopy, NBI, iSCAN, FICE, CLE)

## 

**Video 1** Setup of the dual-pump switchable irrigation system from saline irrigation to gel immersion, and clinical use during water pressure endoscopic submucosal dissection for a large duodenal epithelial tumor.


The water pressure method for duodenal endoscopic submucosal dissection (ESD) is performed under saline irrigation, which provides a clear operative field and stable maneuverability. 1 However, when active bleeding occurs, blood rapidly spreads through the saline, resulting in immediate loss of the endoscopic view and making hemostasis difficult.

Gel immersion endoscopy is useful for securing the visual field during gastrointestinal bleeding because gel does not immediately mix with blood and can create a transparent working space. 2 3 This property is also useful during ESD-related bleeding. However, switching from saline irrigation to gel injection generally requires manual injection through the accessory channel or an irrigator-equipped forceps plug. This process may require assistance, interrupt the procedure, and introduce air bubbles through device manipulation. Conversely, continuous gel immersion throughout water pressure ESD may also increase gel consumption, preparation time, and procedural cost. Therefore, a method that allows selective gel use only during bleeding would be desirable.

To overcome these limitations, we developed a dual-pump saline-to-gel switching system ( Fig. 1 , Video 1 ). One flushing pump was used for routine saline irrigation during underwater ESD, while a second pump was connected to a gel bag. The two lines were connected to the auxiliary water channel using a three-way stopcock and check valves, allowing immediate switching between saline and gel by pedal operation.

**Fig. 1:**
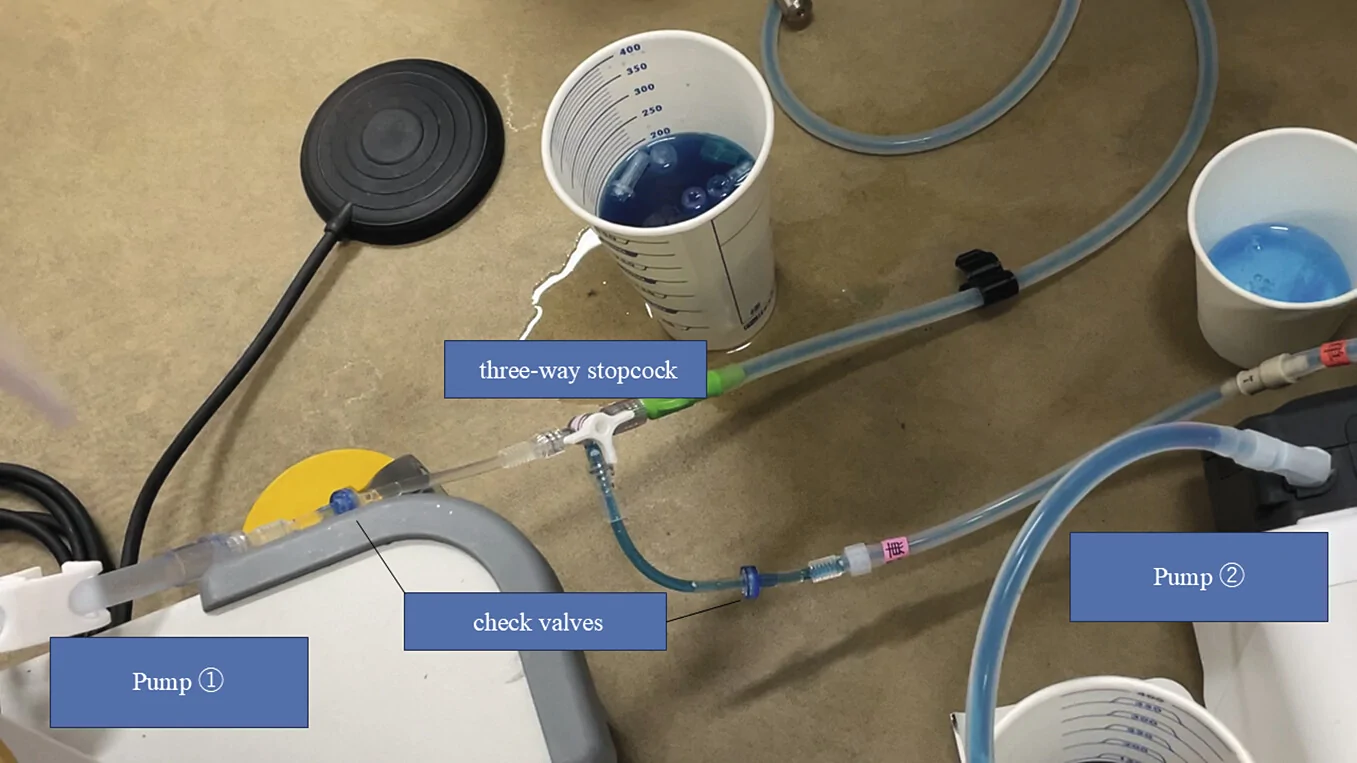
Setup of the dual-pump switchable irrigation system. Two flushing pumps were connected through a three-way stopcock and check valves, allowing selective delivery of saline or gel to the auxiliary water channel. Check valves were placed to prevent air contamination and backflow into the irrigation lines.

With this system, saline irrigation was used during routine dissection, and gel injection was activated when bleeding impaired the visual field. The gel displaced blood and allowed prompt hemostasis without device exchange or air contamination ( Figs. 2 and 3 ). Following successful hemostasis, the lesion was resected en bloc and histopathologically confirmed as a tubular adenoma with R0 resection.

**Fig. 2:**
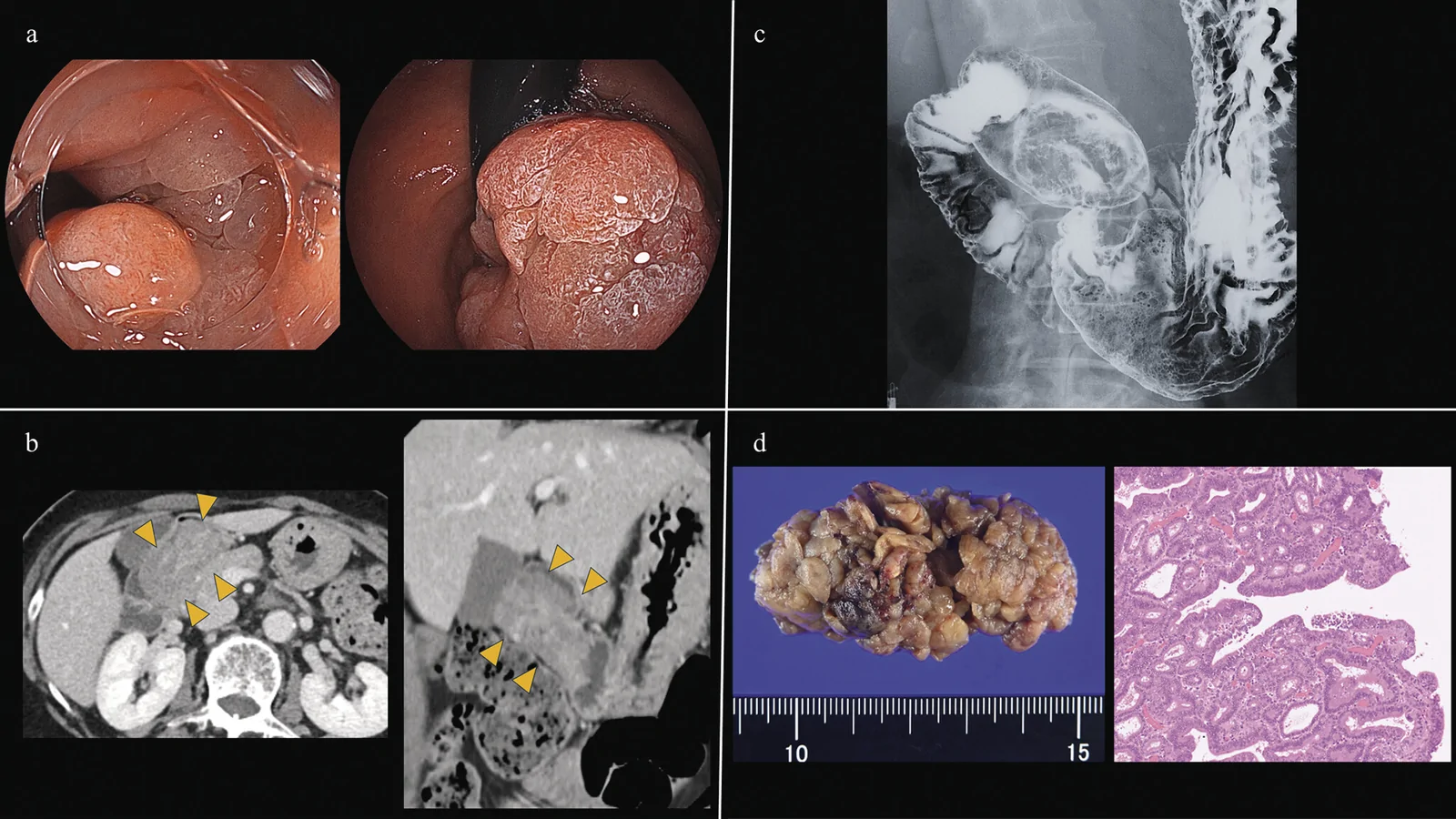
Representative case treated by underwater endoscopic submucosal dissection using the dual-pump switchable irrigation system. A 60-mm elevated lesion was located in the duodenal bulb. The lesion was completely resected without any complications. ( **a** ) Endoscopic image. ( **b** ) Computed tomography image. ( **c** ) Barium fluoroscopic image. ( **d** ) Images of the resected specimen and representative histological findings.

**Fig. 3:**
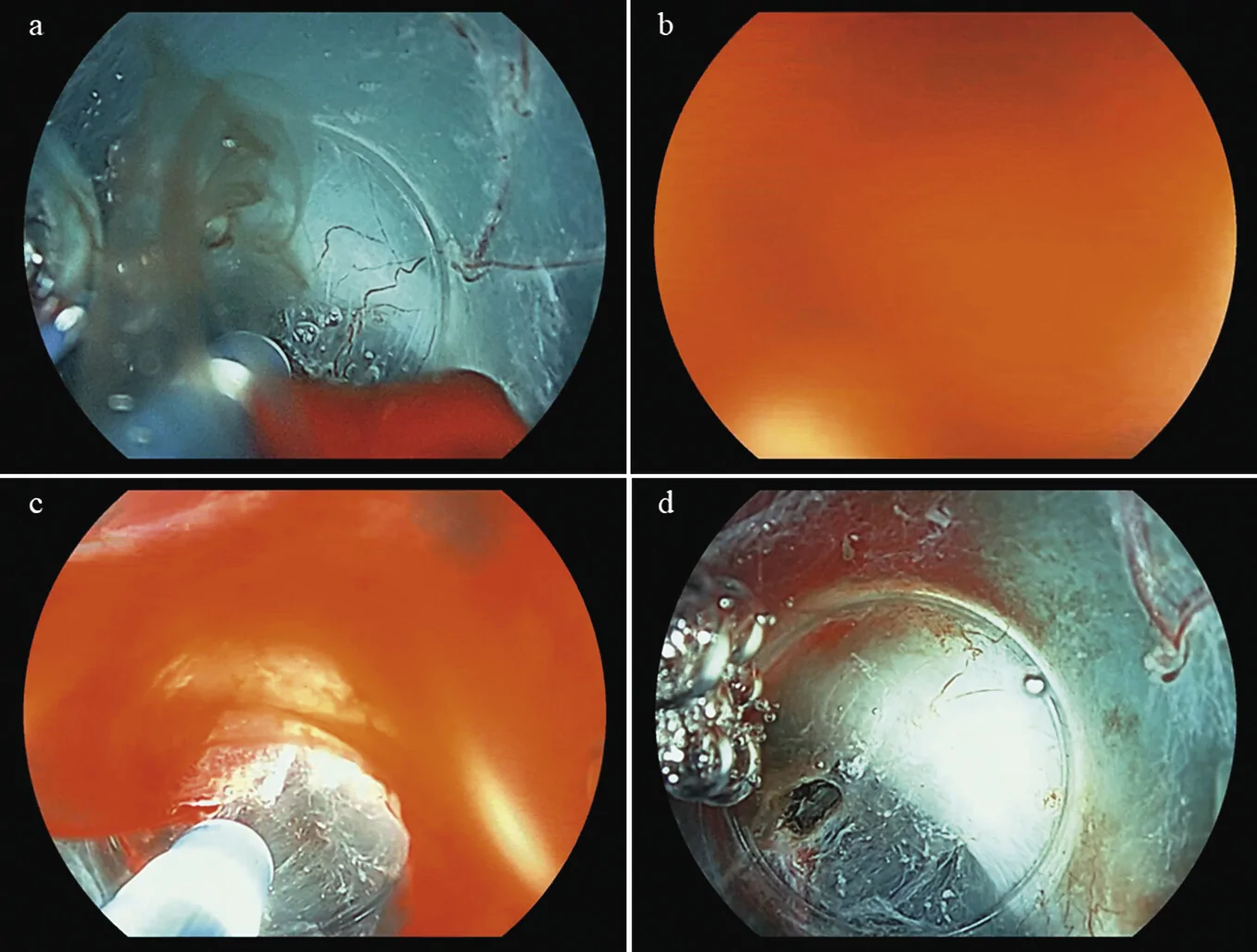
Endoscopic images during spurting bleeding using the dual-pump switchable irrigation system. ( **a** ) A submucosal artery was cut during dissection. ( **b** ) Spurting bleeding occurred, with rapid blood spread in the saline-immersion field. ( **c** ) Irrigation was switched from saline to gel. ( **d** ) Hemostasis was achieved under gel immersion.

This switchable irrigation method may facilitate hemostasis during active bleeding and, through on-demand gel delivery, may also support precise dissection during water pressure ESD.
